# Linking the bed dust microbiome with environmental factors and child respiratory health

**DOI:** 10.1080/03014460.2025.2509606

**Published:** 2025-06-17

**Authors:** Jelena Šarac, Dubravka Havaš Auguštin, Iva Šunić, Kristina Michl, Gabriele Berg, Tomislav Cernava, Damir Marjanović, Rasmus Riemer Jakobsen, Mario Lovrić

**Affiliations:** a Center for Applied Bioanthropology, Institute for Anthropological Research, Zagreb, Croatia; b Faculty of Biotechnology and Drug Development, University of Rijeka, Rijeka, Croatia; c Institute of Environmental Biotechnology, Graz University of Technology, Graz, Austria; d School of Biological Sciences, Faculty of Environmental and Life Sciences, University of Southampton, Southampton, UK; e Burch University, Sarajevo, Bosnia and Herzegovina; f University of Copenhagen, Copenhagen, Denmark; g Lisbon Council, Bruxelles, Belgium

**Keywords:** Dust microbiome, bacterial communities, asthma, child respiratory health

## Abstract

**Background:**

Humans spend up to 90% of their time indoors and are exposed to a significant number of microbes in their homes, which can have important implications for their health.

**Aim:**

This study focused on analysing the influence of environmental factors on microbiome diversity and abundance in bed dust and linking the exposure to dust bacteria with asthma.

**Subjects and methods:**

A total of 90 dust samples were collected from homes of asthmatic patients (*n* = 59) and controls (*n* = 31) aged 5–18 years. The bacterial fraction of the microbiome was analysed using 16S rRNA gene high-throughput sequencing on the Illumina MiSeq platform and downstream analyses in QIIME2 and R. Microbiome profiles were associated with asthma and relevant environmental and household data.

**Results:**

Higher bacterial β-diversity in the environment was shown to be inversely associated with asthma (*p* = 0.009). Also, living environment (*p* = 0.002), housing type (*p* = 0.004), presence of pets in the household (*p* = 0.001), and cleaning practices (*p* = 0.006 for dusting and *p* = 0.011 for vacuuming) were prominent environmental factors affecting the bed dust microbiome.

**Conclusion:**

Our results suggest significant differences in bacterial community composition between individuals with and without asthma and the interaction between indoor microbiome and asthma is mediated by environmental factors in the household.

## 1. Introduction

Individuals in developed countries spend approximately 90% of their time indoors, where they are exposed to a diverse array of microbes within their residential environments, which can have profound health implications (WHO 2024). For instance, exposure to biological agents associated with moisture and mould has been shown to increase the risk of respiratory diseases by 50%. Furthermore, during the COVID-19 pandemic, it was estimated that up to 15% of deaths were attributable to inadequate indoor air quality and poor ventilation (Pozzer et al. 2020). Indoor bacterial communities are significantly influenced by human presence and behaviour, as well as by the outdoor environment. Specifically, the indoor microbiome is shaped by outdoor bacterial communities and the environment in general, including the degree of urbanisation. Urban homes generally exhibit lower microbial richness compared to suburban homes, as demonstrated across multiple studies (Dannemiller et al. 2016; Lehtimäki et al. 2021, 2023; Maestre et al. 2024). Additionally, building materials and housing types contribute to indoor microbial community composition. For instance, wood flooring supports distinct microbial populations compared to synthetic materials, while textiles and firewood enhance bacterial diversity (Coombs et al. 2018; Fu et al. 2020; Cox et al. 2022). Seasonal changes also play a critical role in shaping indoor microbiome diversity, but with a larger impact on the fungal composition. Microbial diversity tends to be reduced during colder months, with bacterial communities predominantly composed of human-associated microbes (Adams et al. 2015; Sitarik et al. 2018). Conversely, warmer and more humid conditions are associated with higher microbial diversity, emphasising the dynamic nature of seasonal effects on indoor ecosystems (Coombs et al. 2018).

Human occupancy and behaviour are additional factors affecting indoor microbial diversity and composition. Spaces with higher occupancy, such as classrooms and multi-member households, exhibit increased bacterial diversity, often dominated by genera like *Staphylococcus* and *Streptococcus*. Frequently used areas, such as children’s bedrooms and living rooms, are particularly affected, while activities like cooking and cleaning were shown to facilitate dispersal of bacteria (Ciaccio et al. 2015; Weikl et al. 2016). Cafeterias and day-care centres were also shown to commonly harbour high bacterial diversity, with cleaning practices significantly influencing microbial abundance (Asif et al. 2018; Coombs et al. 2018; Loo et al. 2018). Furthermore, it has been shown across studies that pets contribute to indoor microbial composition and diversity, especially in farming environments. Pet-associated bacteria, including *Moraxella*, *Porphyromonas*, *Sutterella*, *Clostridium*, and *Mycobacterium*, are more prevalent in pet-owning households. These enriched microbial communities are linked to potential health benefits, such as reduced rates of atopic diseases (Loo et al. 2018; Sitarik et al. 2018; Richardson et al. 2019; Gupta et al. 2020). Overall, studies indicate that pet-owning households harbour higher bacterial loads, which can positively influence indoor air quality and human health (Lee et al. 2021; Cox et al. 2022).

The indoor microbiome has attracted attention in recent years due to its potential impact on respiratory health, particularly in relation to asthma and other respiratory conditions. Numerous studies have investigated this relationship, emphasising the complex interactions between environmental microbial exposures and respiratory health outcomes (Lee et al. 2015; Fu, Li, et al. 2021; Fu, Yuan, et al. 2021; Niemeier-Walsh et al. 2021; Vandenborght et al. 2021; Wang et al. 2023). A significant body of literature has focused specifically on the interactions between environment and respiratory health in children, as the most vulnerable subpopulation for asthma and allergies (Cox et al. 2022; Isa et al. 2022; Sun et al. 2022). Namely, the prevalence and incidence of asthma in recent decades are increasing in children and higher prevalence is observed in countries with higher economic standards. In the Republic of Croatia, the prevalence of asthma ranges between 5% and 8% in children (Čutura 2015). Evidence indicates that specific bacterial taxa present in indoor environments may influence the prevalence and severity of such respiratory conditions. Certain microbial communities have been associated with protective effects, potentially through mechanisms such as modulation of the immune system or competitive inhibition of pathogenic species. Conversely, some other compositions have been linked to adverse respiratory outcomes, likely due to their production of inflammatory agents or allergens that exacerbate symptoms (Fu et al. 2020; Cox et al. 2022). Understanding the underlying mechanisms of microbial dynamics is essential for developing targeted interventions aimed at improving indoor air quality or tailoring microbial exposures to enhance respiratory health and reduce the burden of asthma and related conditions, especially in children as the most vulnerable subpopulation. Continued research is necessary to identify chemical and molecular drivers of these associations and to identify the specific microbial taxa that contribute to either protective or harmful effects.

Despite significant advances in understanding the genetic, microbiome, and immunologic factors influencing asthma, the underlying causes of many phenotypes remain poorly understood (Huang et al. 2017; Fu et al. 2022). Translating theoretical advances into practical prevention strategies remains challenging, with several key issues unresolved. First, a clear epidemiological relationship between the indoor microbiome and asthma, particularly the role of early-life exposure to microbially rich environments, needs further clarification. While many studies have identified health-related indoor microorganisms, no overarching patterns have emerged, making it unclear whether these microorganisms are widely distributed. Additionally, a comprehensive list of protective versus risk-associated microorganisms and metabolites is still lacking, limiting its application for environmental assessment and health outcome prediction. Furthermore, current research often focuses exclusively on one microbial group, particularly bacteria, while neglecting the contributions of the mycobiome and virome and their potential interactions in shaping host immune responses and influencing asthma-related respiratory pathogenesis. Also, environmental factors that affect microbiome diversity, composition, and abundance indoors are not yet fully summarised. Identifying and controlling specific environmental characteristics could offer potential intervention strategies for allergic diseases by fostering beneficial microbial colonisation. Lastly, the molecular targets and mechanisms by which indoor microorganisms influence disease development remain poorly understood, requiring further investigation to identify microbial candidates and therapeutic targets for asthma treatment.

In the attempt to fill a portion of the knowledge gap between indoor microbiome research and understanding the development of asthma in children, we have conducted research of microbiome and environmental factors that could have an influence on children’s respiratory health. The aim of this study was to analyse bacterial bed dust microbiome composition and abundance in a Croatian cohort of asthmatic children and healthy controls.

## 2. Materials and methods

### 2.1. Study cohort and sample collection

**Figure 1.**
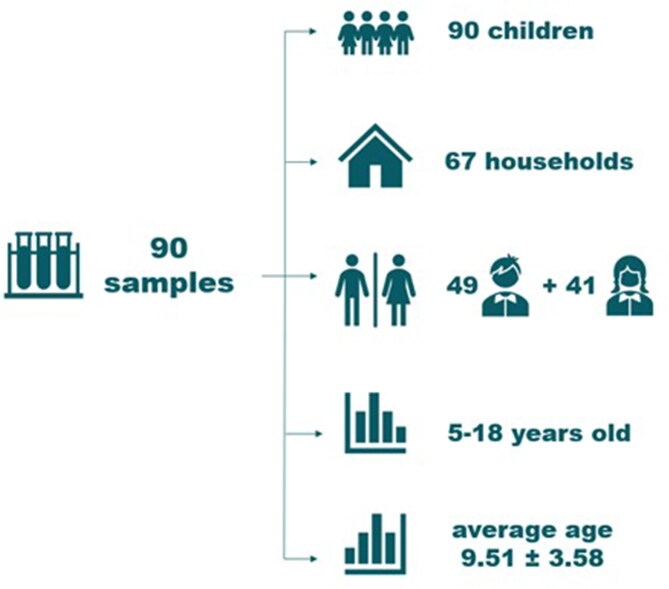
Cohort characteristics.

Overall, 90 samples from the Croatian cohort established within the project “Evidence-driven Indoor Air Quality Improvement—EDIAQI” (www.ediaqi.eu; Lovrić et al. 2025) were included in the study. The cohort’s characteristics are presented in Figure 1. The cohort consists of 59 asthmatic children (65.6%) and 31 children without asthma (34.4%, control group). The diagnosis of asthma was made by an allergology specialist based on: (i) the participant′s medical history and physical examination (including anthropometric measurements), (ii) a significantly positive skin prick test, atopy patch test (APT) or IgE level to inhaled allergens, according to EAACI (European Academy of Allergy and Clinical Immunology) guidelines, and (iii) relevant lung function (spirometry, FENO) and bronchial challenge tests.

Dust samples from children’s bedding were collected using DUSTREAM^®^ Collector vacuum cleaner filters (Indoor Biotechnologies, UK), which were subjected to ultraviolet light irradiation for 30 min prior to use. The filters were affixed to the vacuum cleaner nozzle for the purpose of collecting bed dust, conducted by trained laboratory personnel. Each bed required approximately 5 min of vacuuming of the mattress, pillows, and bedding. Following collection, the filters were stored at −20 °C to preserve microbial genetic material and eliminate dust mites. For microbial DNA extraction, the dust samples were weighed on an analytical scale (Axis ALN120) and aliquoted to 60–80 mg per sample. This specific quantity was found to result in optimal DNA yield.

### 2.2. Microbial DNA extraction

The isolation of genetic material was conducted using the DNeasy^®^ PowerSoil^®^ Pro Kit (Qiagen), specifically developed for the extraction of microbial genomic DNA from a variety of soil types, as well as challenging samples such as sediment and dust. The isolation procedures were performed in accordance with the manufacturer’s protocol. The concentration of the extracted genomic DNA was quantified using the Qubit 3.0 Instrument using the Qubit dsDNA BR Assay Kit (Invitrogen, Thermo Fisher Scientific), yielding concentrations ranging from 5.24 to 135 ng/µL. The isolated DNA was subsequently stored at −20 °C for downstream laboratory analyses.

### 2.3. Library preparation and sequencing

Library preparation was conducted in accordance with the Illumina 16S Metagenomic Sequencing Library Preparation protocol. The diluted samples underwent amplification using the Agilent SureCycler 8800 (Agilent Technologies) with specific primer sets targeting bacterial DNA. The variable regions V3–V4 of the 16S rRNA gene were amplified using primers 341 F and 805 R, as previously reported by Gupta et al. (2020). PCR reactions were performed using KAPA HiFi HotStart Ready Mix (Roche, Basel, Switzerland), with template-free PCR-grade water serving as a negative control and ZymoBIOMICS Microbial Community DNA Standard (Zymo Research, Irvine, CA, USA) as a positive control. The PCR protocol consisted of an initial denaturation step at 95 °C for 3 min, followed by 30 cycles of denaturation at 95 °C for 30 s, annealing at 55 °C for 30 s and extension at 72 °C for 30 s, concluding with a final extension at 72 °C for 5 min. The quality and size of the PCR products were verified using the 4200 TapeStation System (Agilent Technologies). Indexing was performed using sequencing adapters from the Nextera XT Index Kit v2 (Illumina). In the final step, pooled libraries were denatured with 0.2 N NaOH and adjusted to a final concentration of 8 pM, with 5.0% PhiX included as an internal control. Sequencing was performed using the MiSeq Reagent Kit v3 (600 cycles) on the Illumina MiSeq System (Illumina Inc., CA, USA).

### 2.4. Sequence analysis and statistics

The obtained 16S rRNA sequence reads were further processed using well-established bioinformatic pipelines. After demultiplexing, a quality control step was performed where low-quality and chimeric sequences were eliminated and amplicon sequencing variants (ASVs) and a feature table were generated using the DADA2 algorithm within the QIIME2 platform, providing a high-resolution view of the microbial communities in the samples. The ASVs were subsequently classified using the vsearch algorithm and with the latest SILVA v132 database.

Data transformation, statistical analysis, and microbial community profiling were conducted in R [version 4.3.0], primarily using the phyloseq (McMurdie and Holmes 2013), ggplot2 (Wickham 2016), rabuplot (Stokholm 2024), microeco (Liu et al. 2021), and vegan (Oksanen et al. 2022) packages. After collapsing the dataset to summarise ASVs as their corresponding genera, taxonomic abundance was analysed at the genus level. We used PERMANOVA (adonis2) with Bray-Curtis and Jaccard distance metrics to assess variation in bacterial community composition. Barplots were generated to illustrate the overall relative abundance of taxa, while violin plots display the relative abundance distributions for individual taxa. The violin plots include statistical analyses, with taxa that show statistically significant differences (*p* < 0.05) highlighted in bold. Redundancy analysis (RDA) was conducted to assess the relationship between microbial community composition and environmental metadata. Variables explaining more than 20% of the variance were included, and the lengths of arrows were scaled to reflect their explanatory power. The statistical tests used by the function depended on the structure of the data and the number of categories in the predictor. In the case of two groups (pet ownership variable: “Yes” vs. “No”), non-parametric tests such as the Wilcoxon rank-sum test were used, while for comparisons involving more than two groups the Kruskal-Wallis test was used. The Holm-Bonferroni method was applied for multiple testing correction to ensure the reliability of results when analysing multiple microbial taxa simultaneously. Statistical significance was defined as *p* < 0.05 after adjustment. In the end, these statistical methods were integrated into the visualisation workflow which shows comprehensive and precise assessment of the effects of categorical variables on microbial community abundance. For beta diversity analysis, the dataset was normalised using cumulative sum scaling, and Bray-Curtis dissimilarity matrices were calculated. Significant differences between groups were assessed using the adonis2 function (PERMANOVA, 999 permutations) from the vegan package, used for ecological analysis (Oksanen et al. 2022).

### 2.5. Covariates

Information on environmental factors and occupant behaviour was collected through questionnaires completed by the parents. These questionnaires were developed by trained psychologists from the Institute for Anthropological Research and validated on a representative sample. The living environment was categorised into suburban, urban built areas (city, within a built environment), and urban green areas (city, surrounded by green spaces). A distinction was also made between housing types – house vs. apartment. In the category Pets, 97% of participants with pets had a dog or a cat. Dusting furniture was categorised into three categories: occasionally, once a week, and frequently (four or more times per week). Vacuuming mattresses was categorised into never or once a year, two to four times a year, and more than 4 times a year.

## 3. Results

Basic information for the cohort is presented in Table 1. Asthma prevalence was recorded at 65.6%, with 34.4% of healthy individuals. Gender distribution was fairly even, with 45.6% female and 54.5% male participants. Age groups included pre-schoolers (36.67%), school children aged 8–12 (38.89%), and teenagers (24.44%). Asthma prevalence was higher among males (37.78%) than females (27.78%) and most common among children aged 8–12 (30.0%). Parental education levels varied, with most parents having a high education (53.3%), followed by middle-level education (25.6%) and parents with a PhD (21.1%). Household income showed that the majority (64.4%) earned more than 2500 € on a monthly basis.

**Table 1.** Basic cohort information.

Category	Variable	Total, *n* (%)
Asthma	No	31 (34.4)
	Yes	59 (65.6)
Gender	Female	41 (45.6)
	Male	49 (54.5)
Age	Preschoolers (5–7)	33 (36.67)
	Children (8–12)	35 (38.89)
	Teenagers (13–18)	22 (24.44)
Asthma per gender	Female	25 (27.78)
	Male	34 (37.78)
Asthma per age	Preschoolers (5–7)	16 (17.78)
	Children (8–12)	27 (30.0)
	Teenagers (13–18)	16 (17.78)
Education parents	Middle	23 (25.6)
	High	48 (53.3)
	PhD	19 (21.1)
Household monthly income	< 1500 €	7 (7.8)
	1500 €–2500 €	25 (27.8)
	> 2500 €	58 (64.4)

**Figure 2.**
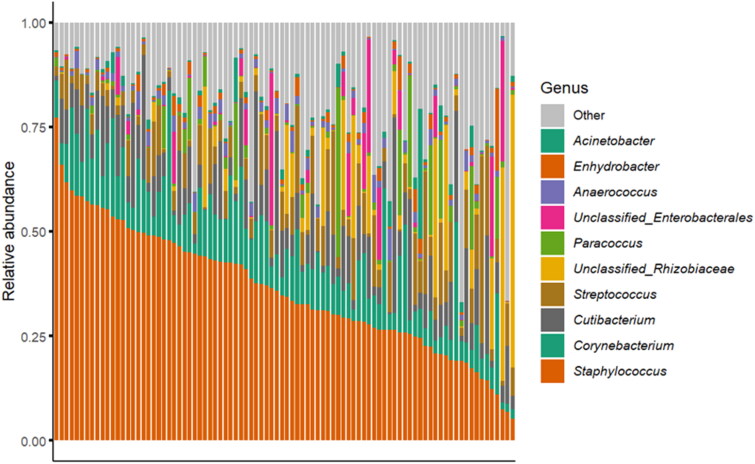
Taxonomic bacterial abundance at the genus level.

Regarding sequencing data, after quality filtering and removal of unassigned taxa, 6,164,389 reads were used for downstream analysis. A total of 16,010 ASVs were identified, distributed across 36 bacterial phyla. Genera with combined abundance below 1% were categorised as “other.” Figure 2 displays the taxonomic composition of samples at the genus level, illustrating the relative abundance of various microbial genera. The most prominent genera were identified as *Staphylococcus* and *Corynebacterium*, along with a range of other less abundant genera, such as *Cutibacterium*, *Streptococcus*, and *Paracoccus*.

### 3.1. Relation to asthma

**Figure 3.**
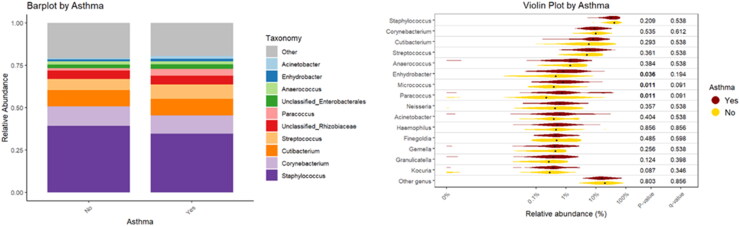
Relative abundance of bacterial taxa with respect to asthma.

A significant part of the variation in the bacterial community composition was explained by the factor asthma (R^2^ = 0.015; *p* = 0.009). This suggests there are notable differences in the bed dust microbial community composition between individuals with and without asthma. Bacterial communities in the bed dust of individuals with and without asthma showed a consistent distribution of dominant taxa, including *Staphylococcus*, *Corynebacterium*, and *Cutibacterium*, across both groups. However, subtle differences in the abundance of less prevalent taxa were found, as evident in the violin plot. Notably, *Enhydrobacter* (*p* = 0.036), *Micrococcus*, and *Paracoccus* (*p* = 0.011 for both) showed statistically significant differences in abundance between the two groups (Figure 3).

### 3.2. Environmental influences

**Figure 4.**
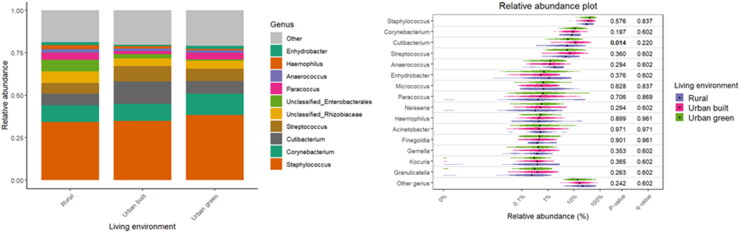
Relative abundance of bacterial taxa with respect to the living environment.

**Figure 5.**
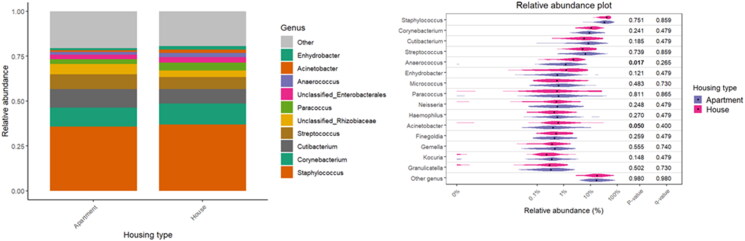
Relative abundance of bacterial taxa with respect to the housing type.

**Figure 6.**
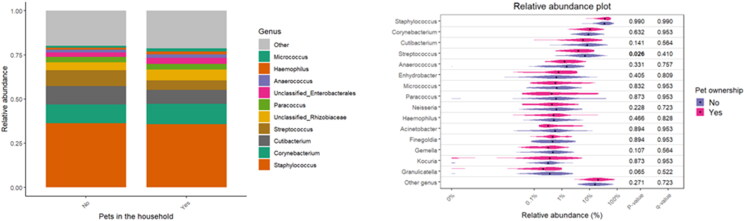
Relative abundance of bacterial taxa with respect to pet ownership.

Specific associations between the diversity and relative abundance of microbial taxa with living environment, pets, and certain occupant practices have been observed. Table 2 provides insight into the household’s potential influence on the composition of bacterial communities (β-diversity). The living environment was shown to significantly influence bacterial β-diversity (R^2^ = 0.029; *p* = 0.002), along with a notable interaction with asthma (R^2^ = 0.030; *p* = 0.001). The type of home has also shown significant associations with β-diversity of the bacterial community (R^2^ = 0.016; *p* = 0.004), but not with asthma. Although the overall microbial community composition was largely stable across different categories, with dominant taxa such as *Staphylococcus*, *Corynebacterium*, and *Cutibacterium* consistently abundant, subtle variations in less dominant taxa are observed in the violin plots (Figures 4–6). For the living environment (rural vs. urban), *Cutibacterium* has shown notable differences (*p* = 0.014), while *Anaerococcus* and *Acinetobacter* were significantly different between houses and apartments (*p* = 0.017 and *p* = 0.050, respectively). Pet ownership, especially dog and cat ownership (97% of households with pets), had a marked effect on the bacterial fraction of the microbiome, with significant associations for bacterial β-diversity (R^2^ = 0.019; *p* = 0.001) and asthma interaction (R^2^ = 0.014; *p* = 0.021). Regarding bacterial abundance, *Streptococcus* has been found to be significantly more abundant in households without pets, with a *p*-value of 0.026.

**Table 2.** Influence of household factors on bacterial community composition and interaction with asthma.

Category	Variable	Total (n, %)	16S	
			β diversity (R2/p-value)	β diversity interaction with asthma (R2/p-value) (N = 59)
Living environment	Suburban	19 (21.1)	0.029/0.002*	0.030/0.001*
	Urban (built surroundings)	35 (38.9)		
	Urban (green spaces)	36 (40.0)		
Type of home	House	30 (33.3)	0.016/0.004*	0.012/0.190
	Apartment	60 (66.7)		
Pet ownership	No	61 (67.8)	0.019/0.001*	0.014/0.021*
	Yes	29 (32.2)		

* *p*-value < 0.05.

**Figure 7.**
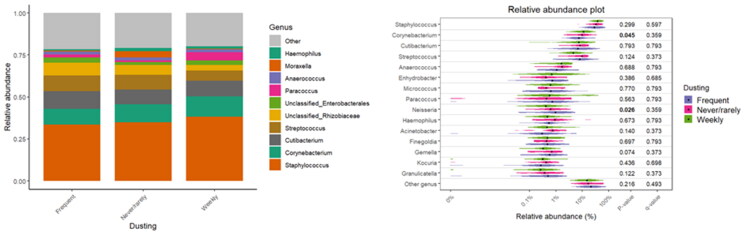
Relative abundance of bacterial taxa with respect to dusting frequency.

**Figure 8.**
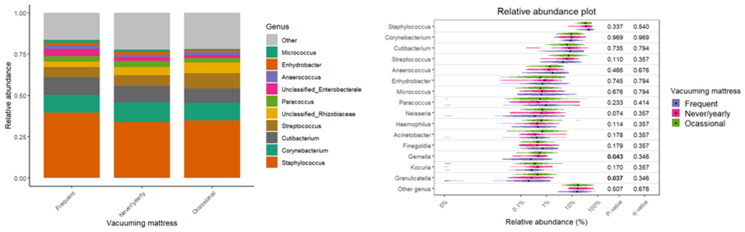
Relative abundance of bacterial taxa with respect to mattress vacuuming frequency.

The results also indicated diversity and relative abundance differences of bacterial taxa based on cleaning practices, namely mattress vacuuming and dusting frequency (Table 3). Dusting furniture has been shown to be significantly associated with bacterial β-diversity (R^2^ = 0.040; *p* = 0.006), with an interaction observed between dusting and asthma (R^2^ = 0.042; *p* = 0.003). Mattress vacuuming is also connected with significant associations for both β-diversity and asthma interaction (R^2^ = 0.027; *p* = 0.011 and R^2^ = 0.028; *p* = 0.002, respectively). Although consistent microbial composition has been observed across different frequencies of mattress vacuuming and dusting and dominant taxa such as *Staphylococcus*, *Corynebacterium*, and *Cutibacterium* remained stable across all groups, the violin plots, used to visualise the distribution of specific taxa, provided finer detail and highlighted specific genera with statistically significant differences (Figures 7 and 8). For dusting, *Neisseria* showed significant differences (*p* = 0.026), alongside *Corynebacterium* (*p* = 0.045). For mattress vacuuming, a significant association between the abundance of *Gemella* (*p* = 0.043) and *Granulicatella* (*p* = 0.037) and mattress vacuuming has been observed.

**Table 3.** Influence of cleaning practices on bacterial community composition and interaction with asthma.

Category	Variable	Total (n, %)	16S	
			β diversity (R^2^/p-value)	β diversity interaction with asthma (R^2^/p-value) (N = 59)
Dusting furniture	Occasionally	15 (16.7)	0.040/0.006*	0.042/0.003*
	Once a week	45 (50.0)		
	Several times a week	30 (33.3)		
Mattress vacuuming	Never or once a year	19 (21.1)	0.027/0.011*	0.028/0.002*
	2–4 times a year	43 (47.8)		
	< 4 times a year	28 (31.1)		

* *p*-value < 0.05.

While statistically significant associations are observed, the R^2^ values in many PERMANOVA analyses are low, which is common in microbiome studies due to the complexity of microbial communities and multiple influencing factors. Microbial composition is highly multidimensional, shaped by environmental, host-associated, and stochastic elements, meaning each factor, like asthma, explains only a small fraction of variance. High inter-individual variability, even within the same environment, further reduces R^2^ values. Moreover, the microbiome variability in dust is shaped by multiple interacting factors, making it difficult to isolate the effect of asthma status alone. Study design, beta diversity metrics, small sample size or high within-group variability can also obscure between-group differences, limiting the explanatory power of statistical models and contributing to low R^2^ values. Nevertheless, even small microbial shifts can have biologically meaningful implications for health outcomes like asthma. Since environmental factors shaping the indoor microbiome are adaptable to a certain degree, they present a valuable opportunity for interventions and prevention strategies aimed at improving respiratory health.

**Figure 9.**
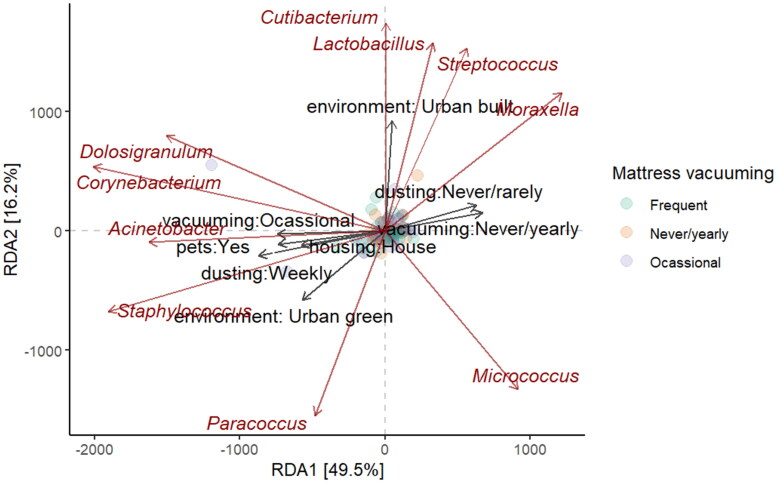
RDA analysis for bacterial genera.

To understand the drivers of variation in indoor bacterial communities in more detail, a redundancy analysis (RDA) was conducted, as presented in Figure 9. The plot revealed that cleaning practices, pets, and living environment collectively explain a significant proportion of the variation in bacterial community composition. The latent variable RDA1 (49.5%) captures the primary axis of variation in bacterial communities, influenced heavily by cleaning practices and living environment. RDA2 (16.2%) represents secondary variation driven by household characteristics like housing type and pets, as well as cleaning practices. Bacterial genera displayed distinct associations with certain environmental variables. Based on the RDA plot, *Streptococcus* and *Moraxella* thrive more in urban, built environments and households where cleaning is less frequent, while *Staphylococcus*, *Acinetobacter*, and *Corynebacterium* are associated with households where cleaning practices are moderate to frequent, suggesting that they primarily derive from human inhabitants and are negatively associated with urban green environments. *Acinetobacter* was also more prevalent in households with pets.

## 4. Discussion

A comprehensive understanding of indoor microbiomes and their determinants is essential to decipher how modern, human-built environments affect our health. This study represents a pilot, offering insights into the effects of environmental factors on the bacterial fraction of the microbiome present in bed dust in a Croatian cohort, and its relation to asthma in children. This is the first study of this kind conducted in Croatia, addressing a pressing public health issue. Namely, the prevalence of asthma in Croatia is 5.0% of the total population or approximately 200,000 people and higher among children (up to 8%) (Čutura 2015; Croatian Public Health Institute 2022).

The detected association between bacterial β-diversity and childhood asthma highlights notable differences in microbial community composition between children with and without asthma. This suggests a potential link between asthma and alterations in bacterial communities indoors. A set of environmental characteristics, such as living environment, pet ownership, and cleaning practices, have also been shown to play a substantial role in shaping bacterial β-diversity. Additionally, the observed significant interaction between these factors and asthma implies that environmental factors may mediate the relationship between microbial β-diversity and childhood asthma and highlights the complex relationship between bacterial community composition, environmental exposures, and respiratory health in children.

The genera identified in this study represent a diverse array of microorganisms commonly associated with human, animal, and environmental microbiomes. Genera such as *Staphylococcus*, *Corynebacterium*, *Cutibacterium*, *Streptococcus*, and *Anaerococcus* are typical residents of human skin, mucosal surfaces, or the salivary microbiome, suggesting the likely contribution of human activity and the skin microbiome to the dust microbiome in indoor environments. The abundance of *Staphylococcus* in asthma-associated environments aligns with prior studies linking its abundance to asthma-related conditions (Bachert et al. 2020; Chen et al. 2022). Although *Staphylococcus* is a common coloniser of humans, it can become a dangerous pathogen under favourable conditions. For example, *Staphylococcus aureus*, as a frequent inhabitant of the upper airways, has been linked to triggering allergies and inducing IgE production and higher rates of *S. aureus* colonisation and sensitisation to its proteins have been observed in conditions such as atopic dermatitis, nasal polyps, and asthma (Bachert et al. 2020).

Environmental genera, including *Paracoccus* and *Acinetobacter*, highlight the role of soil in shaping the indoor microbial landscape, likely through tracked-in dirt or airborne particulates. *Paracoccus* is a bacterial genus known for its metabolic diversity and its ability to thrive in both unpolluted and anthropogenically shaped environments (Decewicz et al. 2019). *Acinetobacter* is also one of the “common to all” core house dust microbiome components, although there are certain strains that can cause infections or pneumonia (Doughari et al. 2011).

The relative abundance distributions for individual taxa highlight specific bacteria that drive the difference between households with regards to different environmental factors. Suburban, urban-green, and urban-built environments vary regarding the abundance of the *Acinetobacter* bacteria, suggesting that access to greenery or proximity to built environments influences microbial exposure. Such findings support previous research indicating that urbanisation alters microbial diversity, often reducing beneficial outdoor-derived taxa (Barberán et al. 2015; Shan et al. 2019). Housing type is also closely connected to the urbanisation level, suggesting similar results – households in urban areas are more often apartments, while households in rural areas are more often family houses, harbouring increased bacterial richness (Dannemiller et al. 2016; Maestre et al. 2024). A study conducted by Fu, Ou, et al. (2021) assessed the health effects of microbial exposure among high schoolers and the overall indoor microbiome taxonomic and functional composition was significantly different between urban and rural schools, with more potential pathogens in urban schools. The presence of pets was also associated with certain bacterial taxa (*Streptococcus* and *Acinetobacter*), which is in line with previous studies showing that specific bacterial composition and increased diversity in general are associated with the presence of pets in the household, with a protective influence on health (Kettleson et al. 2015; Liu 2015; Hickman et al. 2022). A study conducted on two birth cohorts by Mäki et al. (2021) suggested that living with dogs may help protect children against allergic diseases and airway infections, potentially due to immunomodulation from microbial exposure associated with dogs. Hickman et al. (2022) also analysed dust samples from homes of families with young children and observed significantly larger bacterial richness in homes with dogs than in homes without a dog.

The findings of this study also emphasise the significant impact of cleaning practices on the microbial diversity and composition of household dust, highlighting taxa such as *Staphylococcus* as predominant in households where cleaning practices are more frequent and *Streptococcus* being more abundant in households where cleaning practices have been reported as very rare. These findings align with earlier research suggesting that frequent cleaning may selectively alter microbial profiles, often reducing diversity and potentially diminishing beneficial microbial exposures (Barberán et al. 2015; Shan et al. 2019). This is also in line with our finding that households with asthmatic children have increased relative abundance of *Staphylococcus*, because such households also report increased frequencies of dusting and mattress vacuuming. Specific taxa such as *Neisseria* also showed significant differences across dusting categories, consistent with studies indicating that frequent cleaning practices favour such resilient microbes (Nygaard and Charnock 2018). It is important to stress that reduced microbial diversity due to frequent dusting may have implications for immune tolerance, particularly in children, as exposure to a diverse range of microbes in early life is linked to lower asthma risk (Ege et al. 2011). The study findings regarding cleaning practices in general are in line with the “hygiene hypothesis,” which suggests that reduced microbial exposure due to rigorous cleaning practices may impair immune development and increase susceptibility to asthma and other allergic conditions in children (Ege et al. 2011; Shan et al. 2019). Namely, the general rise in allergies in the last few decades contrasts with the reduced prevalence of infectious diseases due to improved public health measures, medical treatments, vaccinations, and hygiene. This has also led to decreased exposure to non-infectious microorganisms, which has been proposed as a possible explanation for the allergy and asthma epidemic. The “hygiene hypothesis” is based on evidence that overcrowding, poor hygiene, and larger family sizes were linked to lower rates of atopy, eczema, hay fever, and asthma, so increased early-life exposure to infections and microorganisms was suggested as a protective factor (Brooks et al. 2013).

## 5. Conclusions

This study highlights the significant influence of environmental factors on the β-diversity and abundance of the indoor microbiome in household dust, and its association with respiratory health in children. The detected association between bacterial β-diversity and asthma highlights differences in microbial community composition between individuals with and without asthma, suggesting a potential link between asthma and alterations in the bacterial microbiome. Certain environmental determinants, including living environment, housing type, pet ownership, and cleaning practices, were also shown to shape the bacterial composition of indoor dust. Dominant bacterial genera, such as *Staphylococcus* and *Corynebacterium*, remained consistent across samples, while less abundant taxa demonstrated variability linked to specific environmental factors. These findings highlight the complex interplay between microbial β-diversity, environmental exposures, and respiratory health, offering valuable insights into strategies for improving indoor air quality and promoting respiratory health. Future research should aim to expand cohort sizes and explore the mechanistic pathways through which specific microbial taxa influence asthma and related conditions, providing a foundation for targeted interventions.

## 6. Strengths and limitations

This study represents a pioneering investigation into the impact of environmental factors on the bacterial microbiome present in household dust within a Croatian cohort of asthmatic children and healthy controls, providing novel insights into largely unexplored health aspects of the microbiome with regards to respiratory health in children. To the best of our knowledge, this is the first study of indoor environmental microbiome not only in Croatia, but in the whole of Southern–Eastern Europe, providing valuable information on microbial communities in this geographical region. However, there are several limitations. The relatively small cohort size introduces potential biases and constraints on the generalisability of the findings, e.g. limited sample size leads to the overrepresentation or absence of certain household characteristics within specific subgroups and it also restricts statistical power, particularly when analysing rare taxa or less common household scenarios, potentially underrepresenting trends or genera that might emerge in larger, more diverse populations. Moreover, even though Illumina sequencing (NGS) has revolutionised microbiome research by providing high-throughput, detailed insights into microbial communities, it comes with some limitations such as the reliance on 16S rRNA gene sequencing for bacterial identification. This method, although widely used, may not provide full taxonomic resolution, especially at the species level. Also, the large amount of data generated by Illumina sequencing requires robust bioinformatics tools which may introduce additional challenges in data analysis and interpretation. Another limitation is that all samples are treated as independent replicates, but certain samples or children come from the same household, which introduces a certain bias. Despite these constraints, this pilot study provides important foundational data and highlights the potential influence of environmental and household factors on microbial communities. Addressing these limitations in future research will be critical for advancing our understanding of indoor microbiome dynamics and their broader implications for human health.

## Data Availability

The authors confirm that additional data supporting the findings of this study will be shared upon reasonable request.
